# MicroRNAs and immunity in periodontal health and disease

**DOI:** 10.1038/s41368-018-0025-y

**Published:** 2018-08-06

**Authors:** Xianghong Luan, Xiaofeng Zhou, Afsar Naqvi, Marybeth Francis, Deborah Foyle, Salvador Nares, Thomas G. H. Diekwisch

**Affiliations:** 1 Department of Oral Biology, UIC College of Dentistry, Chicago, IL USA; 2 Department of Periodontics, UIC College of Dentistry, Chicago, IL USA; 3 Department of Periodontics, Texas A&M College of Dentistry, Chicago, IL USA

## Abstract

MicroRNAs (miRNAs) are critical regulators of the host immune and inflammatory response against bacterial pathogens. In the present review, we discuss target genes, target gene functions, the potential regulatory role of miRNAs in periodontal tissues, and the potential role of miRNAs as biomarkers and therapeutics. In periodontal disease, miRNAs exert control over all aspects of innate and adaptive immunity, including the functions of neutrophils, macrophages, dendritic cells and T and B cells. Previous human studies have highlighted some key miRNAs that are dysregulated in periodontitis patients. In the present study, we mapped the major miRNAs that were altered in our reproducible periodontitis mouse model relative to control animals. The miRNAs that were upregulated as a result of periodontal disease in both human and mouse studies included miR-15a, miR-29b, miR-125a, miR-146a, miR-148/148a and miR-223, whereas miR-92 was downregulated. The association of individual miRNAs with unique aspects of periodontal disease and their stability in gingival crevicular fluid underscores their potential as markers for periodontal disease progression or healthy restitution. Moreover, miRNA therapeutics hold great promise for the future of periodontal therapy because of their ability to modulate the immune response to infection when applied in conjunction with synthetic antagomirs and/or relatively straightforward delivery strategies.

## microRNAs and periodontal disease

Periodontal disease is caused by the host immune and inflammatory response to the bacterial infection of teeth. From a clinical perspective, periodontal disease alternates between episodes of disease activity and episodes of quiescence, and if untreated, progresses from mild inflammation to severe tissue destruction. The periodontal host response to oral bacteria consists of two distinct but related lines of defense, innate immunity and adaptive immunity. Periodontal innate immunity is the first line of defense against invading oral pathogens, which consists of the oral epithelial barrier and the activity of phagocytic cells, such as neutrophils and macrophages, that directly attack and remove invading bacteria. In contrast, adaptive immunity is an antigen (Ag)-specific immune response that depends on the functions of B and T cells. Adaptive immunity is based on the identification and recognition of an Ag on the surface of an infected cell and a subsequent immune response designed to attack the pathogen or infected cell. Together, innate and adaptive immunity collaborate to limit bacterial infection and re-establish periodontal tissue homeostasis.

During the last decade, microRNAs (miRNAs) have emerged as critical regulators of the immune response based on their ability to interfere with the post-transcriptional expression of multiple target genes. miRNAs are short (19–24 nucleotides in length) non-coding RNAs that function either through translational inhibition or mRNA destabilization through sequence-specific binding sites within the mRNA 3′-untranslated region (UTR) of genes. miRNAs affect target gene expression by modulating and fine-tuning protein expression levels rather than switching genes simply on or off.^1,2^ During immune and inflammatory responses, miRNAs target inflammatory regulators and affect the magnitude of the inflammatory response.^3,4^ Recent studies have documented that miRNAs are not only involved in the response against bacterial pathogens but also target a host of other pathogens of viral, fungal and parasitic origin.^5–8^

Many chronic and acute diseases are associated with aberrant miRNA expression levels, which in turn affect gene expression and cellular functions during disease progression. For example, miRNAs are dysregulated in infectious diseases,^5^ autoimmune diseases,^9^ chronic inflammatory diseases,^10^ cardiovascular disorders,^11^ nervous system disorders^12^ and other diseases. Dysregulation of miRNA expression in tissues is reflected in biofluids, such as serum, saliva and crevicular fluid of the gingiva.^13,14^ Therefore, miRNAs may be used as specific and sensitive biomarkers indicative of many diseases. The involvement of miRNAs in various stages during the host response against bacterial infections is highly dependent on the cellular context, with different cell types responding differently to the same pathogen.^15^ The ability to manipulate miRNA expression using gain or loss of function approaches enables selective targeting of miRNA pathways involved in human diseases as a promising strategy for therapeutic interventions against multiple pathological conditions.

## miRNA expression profile changes in gingival tissues of periodontally diseased populations

**Fig. 1:**
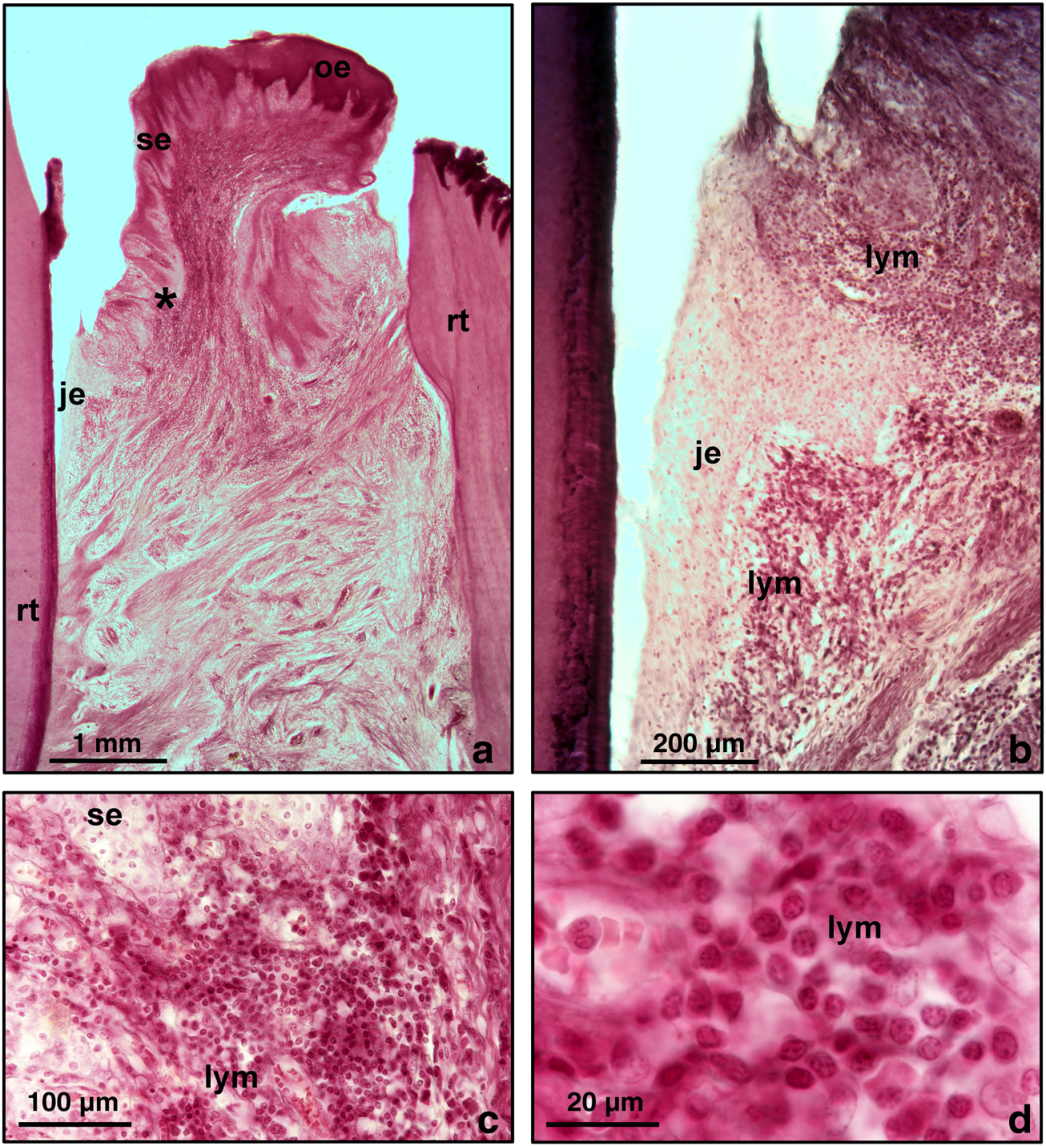
Inflammatory connective tissue infiltrate as a result of chronic inflammatory conditions in an established/advanced periodontal lesion. Micrographs are from freshly fixed human periodontal tissues as preserved in the collection of Joseph-Peter Weinmann at the University of Illinois at Chicago. These slides were originally generated in Gottlieb’s Oral Biology Laboratory at the University of Vienna and transported to Illinois during the 1930s. **a** Overview micrograph illustrating the position of periodontal soft tissues in between two adjacent root surfaces (rt). The periodontal connective tissues are separated from the oral cavity by three distinct types of epithelia, junctional epithelium (je), sulcus epithelium (SE) and oral epithelium (oe). The site of the inflammatory infiltrate is identified with an asterisk (*). Note the relative loss of collagen fibres (**). **b** Most of the leukocyte-rich infiltrate (wbc) is located in the sub-epithelial connective tissue. **c**, **d** Higher magnification micrographs identify individual lymphocytes (lymph) as major components of the inflammatory infiltrate, suggesting that the tissue sample is from a relatively young individual.

**Fig. 2:**
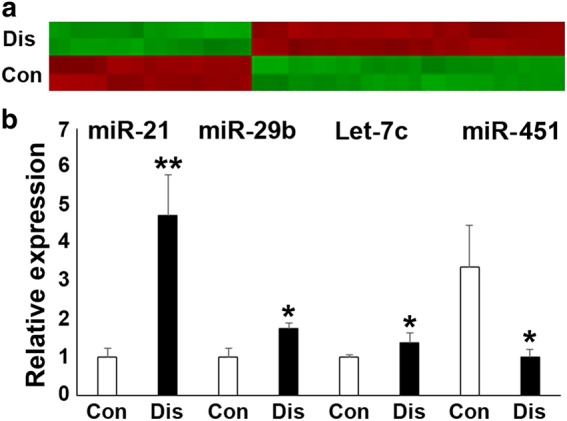
Microarray analysis of microRNA expression in periodontal progenitors from healthy individuals (Con) and animals suffering from periodontal disease (Dis). **a** Heat map of miRNA expression profiling. miRNAs with a significant level of upregulation or downregulation (*P* < 0.01) were identified using Student’s *t* test. Individual upregulated and downregulated genes are listed in Table 1. **b** Quantitative reverse transcriptase (qRT) polymerase chain reaction verification of selected microRNAs in healthy and periodontal disease tissues. There was a significant difference in miR-21, miR-29b, let-7c and miR-451 gene expression between healthy and diseased tissues.

**Fig. 3:**
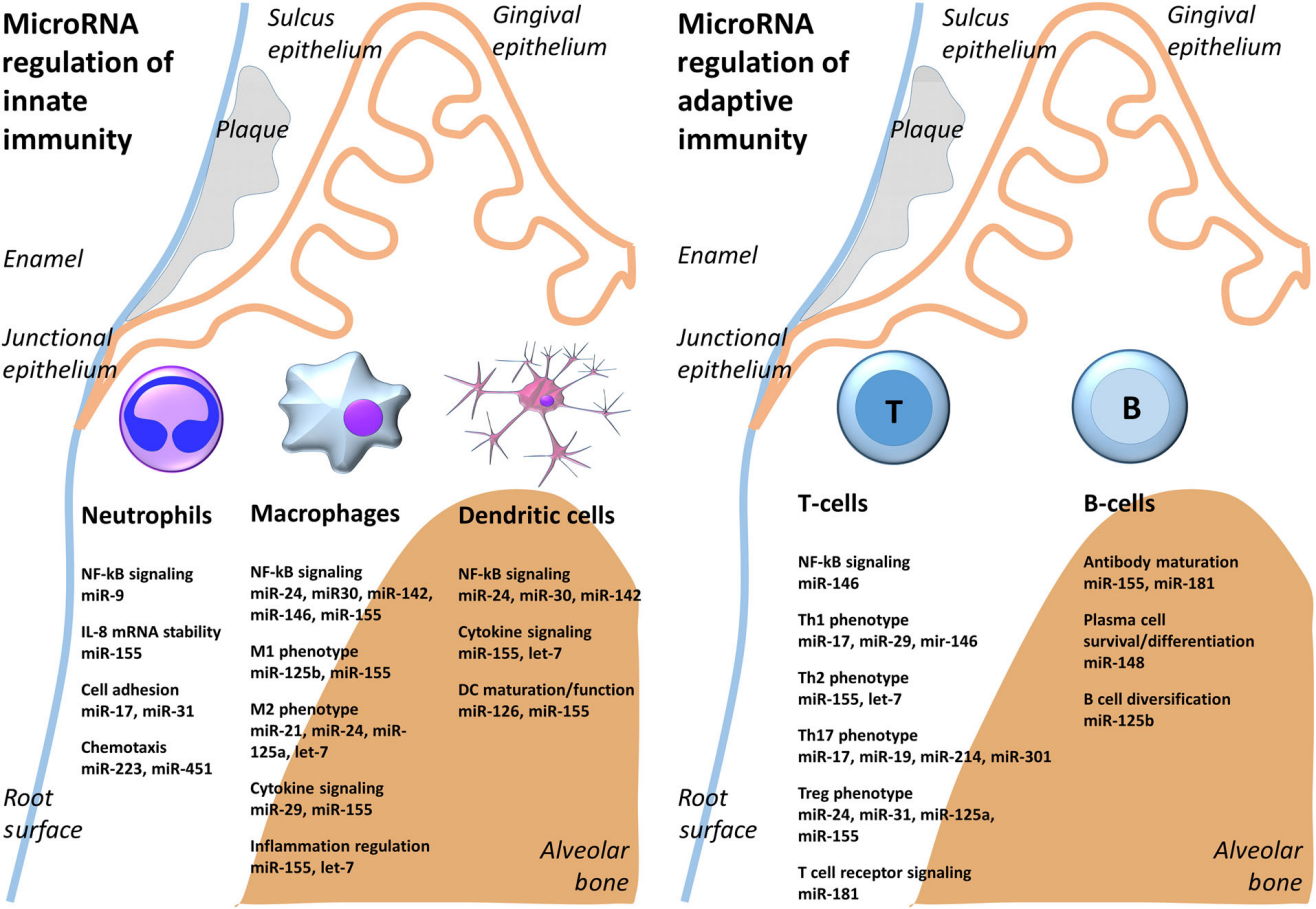
MicroRNA regulation of innate and adaptive immunity in the periodontium. Bacterial plaque on the surface of enamel and in the gingival sulcus induce immune response in the periodontium. By affecting individual target genes, microRNAs either promote or inhibit the function of innate immune cells including neutrophils, dendritic cells and macrophages, and/or the function of adaptive immune cells including T and B cells.

The gingiva is a part of the oral mucosa and consists of dense connective tissue and overlying oral epithelium. Gingival tissues are inhabited by a wide variety of microbes, including commensal organisms; however, they also have a high level of susceptibility to continuous attacks by oral pathogens because of their unique anatomic position between the oral biofilm and the underlying connective tissues of the periodontium. In healthy environments, the gingival microenvironment is inhabited by unique subsets of immune cell populations. When exposed to inflammatory conditions, immune cells infiltrate the oral epithelium and its underlying connective tissue (Fig. 1). Regulation of the immune-inflammatory response greatly affects patient susceptibility to periodontal disease. To determine whether miRNAs are involved in the regulation of the immune response during periodontitis progression, we performed miRNA expression profiling analysis using our periodontitis animal model. For our analysis, gingival crevices from healthy and diseased animals were dissected, and miRNA expression profiles were compared using a microRNA microarray (LC Sciences, Houston, TX, USA). Our profiling study demonstrated significant changes in miRNA expression in gingival tissues from control and periodontitis animals (Figs. 2 and 3). Some of the upregulated or downregulated miRNAs are listed in Table 1.

**Table 1:** Changes in miRNA expression profile in gingival tissues of periodontitis animals compared to control animals

miRNAs	Change in expression	miRNAs	Change in expression
miR-15a	Up	miR-17	Down
miR-21a	Up	miR-24	Down
miR-26a	Up	miR-30	Down
miR-29b	Up	miR-92a	Down
miR-126a	Up	miR-451	Down
miR-125a	Up		
miR-146a	Up		
miR-146b	Up		
miR-148a	Up		
miR-181b	Up		
miR-223	Up		
let-7e	Up		
let-7f	Up		
let-7j	Up		
let-7k	Up		

Recently, several human subject studies have also compared miRNA expression profiles between healthy and diseased gingival tissues^16–19^ or healthy and obese subjects.^20^ Groups of miRNAs that were upregulated or downregulated in tissues with periodontal disease are listed in Table 2. These profiling studies demonstrated that periodontal disease resulted in a changed miRNA expression pattern in diseased tissues. Changes in miRNA levels affect both primary and secondary immune responses of the host against bacterial infection in periodontal tissues.

**Table 2:** Changes in miRNA expression in diseased gingival tissues of periodontitis patients compared to healthy controls

miRNAs	Changes in expression	References	Expression in cells*
miR-9	Up	16, 19	Depleted in epithelial cell
miR-15a	Up	20	Enriched in leukocyte, haematopoietic cell, lymphocyte Depleted in epithelial cell, fibroblast
miR-17	Up	16, 19	Enriched in epithelial cell, endothelial cell
miR-19a,b	Up	16, 19	Enriched in epithelial cell, endothelial cell, haematopoietic cell, leukocyte, monocyte Depleted in fibroblast
miR-21	Up	16	Enriched in myeloid leukocyte, monocyte, phagocyte Depleted in neutrophil
miR-26	Up	16	Enriched in haematopoietic cell, leukocyte Depleted in epithelial cell
miR-29a,b,c	Up	16, 18, 19	Enriched in haematopoietic cell, mononuclear cell, lymphocyte, lymphocyte Depleted in endothelial cell, epithelial cell, fibroblast
miR-30b,c,d	Up	16, 19, 20	Enriched in haematopoietic cell, leukocyte, epithelial cell Depleted in endothelial cell, fibroblast
miR-34a,c	Up	16, 19	Enriched in endothelial cell, epithelial cell Depleted in haematopoietic cell, leukocyte, lymphocyte
miR-125a	Up	16, 19	Enriched in haematopoietic cell, leukocyte Depleted in B cell, meso-epithelial cell
miR-125b	Up	16, 19	Enriched in fibroblast Depleted in haematopoietic cell, leukocyte, lymphocyte
miR-126	Up	18, 19	Enriched in endothelial cell, meso-epithelial cell Depleted in ecto-epithelial cell
miR-142	Up	16, 19, 20	Enriched in haematopoietic cell, leukocyte, lymphocyte Depleted in epithelial cell, fibroblast
miR-146a	Up	19	Enriched in haematopoietic cell, leukocyte, monocyte Depleted in endothelial cell
miR-148	Up	18	Enriched in haematopoietic cell, leukocyte, monocyte Depleted in endothelial cell
miR-155	Up	18	Enriched in haematopoietic cell, leukocyte, monocyte Depleted in endothelial cell
miR-181c	Up	16, 19	Enriched in haematopoietic cell, leukocyte
miR-223	Up	17, 18	Enriched in haematopoietic cell, leukocyte Depleted in epithelial cell
miR-301	Up	16, 18	Enriched in haematopoietic cell, leukocyte Depleted in epithelial cell
let-7b–e	Up	16, 18	Depleted in haematopoietic cell, leukocyte, lymphocyte
let-7f, g	Up	16, 18	Enriched in haematopoietic cell, leukocyte, lymphocyte
miR-31	Down	18	Enriched in endothelial cell Depleted in haematopoietic cell, leukocyte
miR-92a	Down	16	Enriched in B cell, epithelial cell
miR-214	Down	16	Enriched in fibroblast Depleted in haematopoietic cell, leukocyte, epithelial cell
miR-451	Down	16	Enriched in haematopoietic cell, neutrophil, leukocyte Depleted in endothelial cell, epithelial cell

http://fantom.gsc.riken.jp

## miRNA regulation of innate immunity

The oral epithelium and the immune cells residing within periodontal tissues provide the first line of defense against oral bacteria and participate in the primary host response against infection, the innate immune response. The innate immune response is not specifically directed against individual pathogens, but instead provides an initial non-specific defense mechanism during the first hours after infection. From an evolutionary perspective, the innate immune system is a fairly basal defense against pathogens compared to the adaptive immune system as it occurs in invertebrates and vertebrates. In addition to the oral epithelial cells that function as a barrier against toxins and pathogens from the oral cavity, the major cells involved in innate immunity are neutrophils, macrophages and dendritic cells (DCs).

### Neutrophils

Neutrophils are the most important phagocytic cells in the host’s defense  against acute bacterial infection^21^ and the predominant subset of leukocytes in gingival tissues during the early stage of periodontal inflammation. Recent evidence suggests that neutrophils are also involved in the regulation of immunity and inflammation.^22,23^ In neutrophils, miRNAs may act to buffer transcriptional variation and fine-tune gene expression by coordinating the expression of hundreds of transcripts within networks of genes with converging functions.^24^

Our mouse periodontitis model and published human subject studies indicate that several miRNAs expressed in neutrophils were upregulated in diseased periodontal tissues, including miR-155, and miR-223, whereas the neutrophil-expressed miRNAs miR-17 and miR-31 were downregulated (Tables 1 and 2). Here, we discuss two aspects of miRNA involvement in the neutrophil-mediated response to bacterial pathogens and the resulting inflammatory response, (i) the regulation of neutrophil emigration from blood capillaries into inflamed tissues, affecting both neutrophil adhesion to endothelial cells and chemokine mRNA stability, and (ii) the regulation of neutrophil function through miRNAs.

The migration of neutrophils from postcapillary venules into the extravascular tissue at sites of inflammation is one of the first events of the inflammatory response to microbial challenge at the gingival sulcus.^25^ This emigration of leukocytes from the bloodstream into tissues is controlled by intercellular adhesion molecule-1 (ICAM-1) and E-selectin expression, which are targets of the miRNAs miR-31 and miR-17-3p, respectively.^26^ Specific antagonists of miR-31 and miR-17-3p have been reported to increase neutrophil adhesion to cultured endothelial cells, whereas mimics of these miRNAs decreased neutrophil adhesion to endothelial cells.^26^

In periodontal tissues, neutrophil recruitment is dependent upon chemoattractants and their receptors. For example, the CXC motif chemokine receptor 2 (CXCR2) responds to murine analogues of interleukin-8 (*IL-8*).^27^ *IL-8* mRNA stability in neutrophils is modulated by another periodontium-expressed miRNA, miR-155, through the repression of the phosphoinositide lipid phosphatase (*SHIP-1*).^28,29^

Contrary to miR-155, inflammation in periodontitis animals and patients has resulted in a downregulation ofmiR-451 expression (Table 1).^19^ miR-451 directly targets copine 3 (*CPNE3*), Ras-related protein (*Rab5a*) and *14-3-3ζ* genes.^30^ These studies have demonstrated that overexpression of miR-451 affected the phosphorylation of p38 mitogen-activated protein kinase (MAPK) through Rab5a and 14-3-3ζ and suppressed the migration of neutrophils towards *N*-formyl-l-methionyl-l-leucyl-phenylalanine. Systemic application of a miR-451 mimic significantly attenuated the infiltration of neutrophils in an inflammatory animal model.^30^

Neutrophils employ a number of mechanisms to exert an antimicrobial effect on periodontal pathogens. In addition to oxygen-dependent mechanisms, neutrophils rely on granule proteins, including cathepsins, to kill invading bacteria.^22^ Previous studies have demonstrated that miR-223 plays a particularly prominent regulatory role in neutrophils as there are 3819 known proteins affected by the deletion of miR-223 in mouse neutrophils.^24^ miR-223 directly targets the chemokines *CXCL2*, C–C motif chemokine 3 (*CCL3*) and *IL-6* in myeloid cells and controls neutrophil recruitment in chronic inflammatory diseases.^31^ Deletion of miR-223 upregulated the expression of cathepsin family members, suggesting that miR-223 may exert an antimicrobial effect against periodontal pathogens. In addition to the potential protective function against infection, miR-223 is also involved in the activation of nucleotide-binding and oligomerization domain-like receptor containing a pyrin domain 3 (*NLRP3*) inflammasome, which promotes the maturation of inflammatory cytokines IL-1b and IL-18 and induces cell pyroptosis.^32,33^ miR-223 inhibited NLRP3 expression through a conserved binding site within the 3′-UTR and suppressed NLRP3 inflammasome activity.^33^

Nuclear factor-κB (NF-κB) signalling is a proinflammatory signalling pathway and promotes the expression of proinflammatory genes, including cytokines, chemokines and adhesion molecules.^34^ Through NF-κB signalling, miR-9 expression was upregulated in human polymorphonuclear neutrophils and monocytes by the lipopolysaccharides (LPS) of bacterial cell walls after Toll-like receptor (TLR) TLR4, TLR2 and TLR7/8 activation and by treatment with tumour necrosis factor-α (TNF-α) or IL-1β.^35^ However, in these studies, LPS selectively induced only one of three miR-9 genes, *miR-9-1*, in a MyD88-dependent and NF-κB-dependent manner^35^. In neutrophils and monocytes, miR-9 inhibited the expression of *NF-κB1* and operated a feedback regulatory loop, controlling the NF-κB-dependent responses by fine-tuning the expression of a key member of the NF-κB family.^35^

### Macrophages

Macrophages (MΦ) are monocyte-derived large phagocytic cells that play a major role during the innate immune response and contribute to the adaptive immune response by recruiting lymphocytes and other immune cells. In inflamed periodontal tissues, macrophages respond to LPS and activate multiple host defense functions through the production of inflammatory mediators. In turn, the secretion of inflammatory mediators establishes macrophages as the major cellular regulators that cause tissue destruction as a result of periodontal disease.^36^ Both macrophages and DCs are derived from macrophage/DC progenitors (MDPs), a subset of proliferating cells in the bone marrow that share phenotypic characteristics with myeloid precursor populations and give rise to many macrophages and DC subsets.^37,38^ Recent studies have demonstrated that miRNA networks play critical roles in the regulation of macrophage differentiation and activation.^39,40^

To further understand how miRNAs contribute to the differentiation of either macrophages or DCs from monocyte-like macrophage/DC precursors, members of our group have analysed miRNA expression profile changes during monocyte-to-MΦ and during monocyte-to-DC differentiation.^41^ In these studies, parallel profiling of monocyte-to-MΦ and monocyte-to-DC differentiation *in vitro* identified miR-24, miR-30b and miR-142-3p as unique miRNAs that were downregulated in the process of both MΦ and DC differentiation.^41^ All three miRNAs, miR-24, miR-30b and miR-142-3p, appear to counteract periodontal inflammation and inflammation-related tissue destruction. For example, upon challenge with LPS, overexpression of miR-24, miR-30b and miR-142-3p resulted in the suppression of inflammatory cytokine production, including TNF-α, IL-12p40 and IL-6.^41,42^ Transcript analysis of these cytokines revealed that these miRNAs regulate cytokines at both transcriptional and post-transcriptional levels.^42^ Interestingly, intracellular staining of TNF-α demonstrated cytokine accumulation at the cellular periphery of miR-142 mimic transfected cells, suggesting that miR-142-3p may impair cytokine release.^42^ Explaining their anti-inflammatory mode of action, miR-24, miR-30b and miR-142-3p have been demonstrated to target members of the phosphatidylinositol-4,5-bisphosphate 3-kinase (PI3K) and MAPK families and protein kinase C (PKC) isoforms in the TLR4-NF-κB pathway and to inhibit the surface expression of TLR4/cluster of differentiation 14/myeloid differentiation 1 and intracellular PKCα/NFκB activation.^41,42^

miRNAs also modulate the specific macrophage response following exposure to LPS. In one of our studies, LPS derived from periodontal pathogens, *Aggregatibacter actinomycetemcomitans*, *Porphyromonas gingivalis*, or environmentally modified LPS obtained from *P. gingivalis*^43,44^ resulted in LPS-specific miRNA responses in macrophages, including miR-29b and let-7f. miR-29b directly targets interleukin-6 receptor α (*IL-6Rα*) and interferon-γ-inducible protein 30 (*IFI30*), while let-7f targets suppressor of cytokine signalling 4 (*SOCS4*) and thrombospondin-1 (*TSP-1*).^43,44^ These studies demonstrate convergent/divergent miRNA responses to various LPS treatments, and LPS-responsive miRNAs may fine-tune the host response to periodontal pathogens.

In addition to the LPS-response macrophage miRNAs miR-29b and let-7f from our study, the inflammatory miRNAs miR-146 and miR-155 were identified in inflamed gingival tissues^18,19,45^ and are also involved in the activation and function of macrophages.^46,47^ The expression of these miRNAs is NF-κB-dependent and responsive to several TLR ligands in macrophages. miR-146 is considered to be a 'fine-tune' negative feedback regulator in innate immunity. Proinflammatory cytokines IL-1βcytokines as well as LPS up-regulate miR-146 expression through the NF-κB pathway.^47^ miR-146 further inhibits the expression of IL1R-associated kinases (*IRAK1* and *IRAK2*) and TNFR-associated factor (*TRAF6*), major regulatory molecules in the TLR/NF-κB pathway, resulting in a decrease in the expression of NF-κB target genes, such as *IL-1β*, *TNF-α* and type 1 Interferon (IFN).^48,49^ miR-146 also controls the monocyte inflammatory response through the noncanonical NF-κB/Rel pathway by targeting the transcription factor RelB.^50^ However, miR-155 appears to have dual effects on macrophage activation and function. Similar to miR-146, miR-155 directly represses the expression of the inflammatory pathway-related kinase mitogen-activated protein kinase 10 (*MAP3K10*, indicative of  its anti-inflammatory effect on macrophages,^51^ while miR-155 also regulates proinflammatory macrophage activation in a positive fashion by inhibiting the expression of suppressor of cytokine signalling-1 (*SOCS-1*) and increasing type 1 IFN expression.^52,53^ In addition, miR-155 targets B cell lymphoma-6 (*BCL6*) protein and enhances NF-κB signalling.^54^ Taken together, these studies establish miR-155 as a regulatory miRNA that affects various aspects of the inflammatory process dependent on its tissue-specific and stage-related context.

Macrophage polarization as determined by an enhancement in the M1/M2 macrophage phenotype ratio is one of the hallmarks of periodontal disease. Classically activated M1 macrophages are proinflammatory and have a central role in the host defense  against infection, whereas alternatively activated M2 macrophages are anti-inflammatory and participate in tissue remodelling. Changes in the macrophage phenotype ratio are also reflected on the miRNA level. Several miRNAs from our periodontal disease panel (Tables 1 and 2) are involved in macrophage polarization. Among these miRNAs, miR-155 and miR-125b are expressed at a higher level in M1 macrophages than in M2 macrophages and promote M1 polarization.

The mechanism for miR-155 to promote the M1 macrophage phenotype involves suppression of the CCAAT/enhancer-binding protein-β (C/EBP-β) signalling cascade^55^ and targeting of *SHIP-1, IL13Rα1* or *SMAD 2/3* during the regulation of macrophage programming and activation.^46,56–58^ In a similar fashion, miR-125b sustains proinflammatory macrophage activation by targeting the transcription factor interferon regulatory factor-4 (*IRF4*), a positive regulator of alternative macrophage activation.^59,60^ Enforced expression of miR-125b or *IRF4* knockdown enhances macrophage activation, increases the macrophage response to IFN-γ and enhances macrophage-mediated functions, such as stimulating T cell activation and killing mouse tumour cell line EL4 tumour cells.^59,60^

In an antagonistic mode of action to miR-155 and miR-125b, a second group of miRNAs, including miR-125a-5p, let-7c and miR-21, plays an important role in suppressing the classic modes of macrophage activation while promoting alternative activation mechanisms.^61–63^ Illustrating the effect of miRNAs on the M1/M2 macrophage identifier and macrophage function, upregulation of these miRNAs diminished M1 phenotype expression induced by LPS, and promoted M2 marker expression induced by IL-4.^61,62^ In contrast, knockdown of these miRNAs promoted M1 polarization and diminished IL-4-induced M2 marker expression.^61,62^ The negative effect of miR-125a-5p on M1 activation is associated with its target Kruppel-like factor 13 (*KLF13*), a transcriptional factor that has an important role in T lymphocyte activation and inflammation.^62^ The positive effect of let-7c on M2 macrophage polarization is linked to targeting C/EBP-δ, an important transcriptional factor involved in the inflammatory response.^61^ Another miRNA, miR-21, controls macrophage polarization through the cross-talk between miR-21 and the lipid mediator prostaglandin E2 (*PGE2*). In support of this concept, studies have demonstrated that miR-21 directly targets signal transducer and activator of transcription 3 (*STAT3*) and that silencing the *STAT3* gene abolishes the PGE2-mediated expression of M2 genes in miR-21−/− macrophages.^63^ Thus, miR-21 affects M1/M2 ratio values and progression of the immunological macrophage response through PGE2-mediated M2 generation.^63^

Studies from our group have demonstrated that miR-24 is also a negative regulator of macrophage classical activation and promotes alternative macrophage activation.^64^ In these studies, overexpression of miR-24 inhibited the production of *TNF-α*, *IL-6* and* IL-12p40* by macrophages in response to LPS or after exposure to the classic macrophage activation promoter IFN-γ. Moreover, miR-24 overexpression enhanced the expression of *CD206*, a marker of alternative activation, when treated with IL-4 and IL-13. These results indicate that miR-24 promotes an alternative to classical activation as long as macrophage plasticity is maintained, as explained by the miR-24-induced downregulation of the cytokine inducer *p110δ*.

Together, these studies are not only a summary of several strategies by which miRNA expression controls periodontal diseases progression but also a blueprint for future therapeutic approaches towards miRNA-based therapeutics that might interfere with the deleterious sequelae of periodontal disease. From a conceptional point-of-view, overexpression of miR-21, miR-24, miR-125a and/or let-7c during early-stage periodontal disease may inhibit acute inflammation by increasing the number of M2 macrophages.

### Dendritic cells

DCs are professional Ag-presenting cells that reside in periodontal tissues, in other mucosal and lymphoid tissues and in the skin. ^65,66^ The name 'DCs' originates from the tree-branch-like extensions that occur during their development. Originally derived from haematopoietic progenitor cells of the bone marrow, mature tissue-resident DCs are involved in the presentation of Ags and subsequent activation of T cells, killer T cells and B cells through cell–cell contact or cytokine production. During periodontal disease progression, DCs serve as a link between innate and adaptive immunity.^65,66^ The maturation and function of DCs as tissue-resident cells is mediated by the transcription factors c-Fos and purine-rich box-1 (PU.1)^67,68^ and the regulatory molecules SOCS-1 and tuberous sclerosis complex-1 (TSC-1).^69,70^ miRNAs affect primary and secondary immune responses by regulating the expression of these DC-related transcription factors and regulatory molecules.

A number of miRNAs have been identified that regulate DC maturation, development and function. For example, miR-155, miR-126 and Let-7i expression was increased during DC maturation.^71,72^ Moreover, the silencing of *c-Fos* expression or downregulation of *PU.1* by miR-155 modulates the pathogen-binding ability of DCs and promotes Ag-specific T cell activation.^71,73^

Both miR-155 and let-7i target SOCS-1 inside DCs.^74,75^ *SOCS-1*regulates the production of, which is involved in naive CD8 T cell activation.^76^ Downregulation of *SOCS-1* enhances IL-12 production and results in a proinflammatory phenotype in mature DCs.^77,78^ miR-126 directly suppresses the translation of *TSC-1*, a negative regulator of mammalian target of rapamycin (mTOR), and controls the survival and function of plasmacytoid DCs.^79^

Phagocytosis is one of the key functions of the innate immune system and involves the engulfment, degradation and processing of microorganisms, while its foreign Ags are presented to cells of the adaptive immune system.^80,81^ Studies from our group have demonstrated that miR-24, miR-30b and miR-142-3p regulate phagocytosis in DCs and macrophages.^41,82^ Overexpression of miR-24, miR-30b and miR-142-3p significantly attenuated the phagocytosis of *Escherichia coli* (*E. coli*) and *Staphylococcus aureus, as well as* the expression of proinflammatory cytokines (*TNF-α*, *IL-6*, *IL-8* and* IL-12p40*) by macrophages and DCs.^41,82^ Further studies have confirmed that miR-24, miR-30b and miR-142-3p target Fc receptors to regulate antibody-dependent Ag uptake in primary macrophages and DCs.^82^

In our mouse periodontitis model, the expression of a number of DC-related miRNAs, including miR-24, miR-30, miR-126, miR-142, miR-146, miR-155 and let-7i, increased (Table 1), and a similar trend was observed in published studies using human periodontitis models (Table 2). All of these miRNAs were involved in DC and macrophage maturation, as well as Ag processing and presentation by APCs, which is indicative of the role of these miRNAs in the development and progression of periodontal disease.

## miRNA regulation of adaptive immunity

Adaptive immunity is a two-step host response to invading pathogens, beginning with an initial encounter that causes an immunological recognition of a pathogen and followed by an enhanced immunological response upon repeat encounters with the same pathogen. The adaptive immune system is based on the ability of lymphocytes to eliminate pathogens or prevent their growth. In general, adaptive immunity is classified into antibody-mediated immune responses against freely circulating pathogens facilitated by B cells and cell-mediated immune responses against intracellular pathogens that are carried out by T cells. From an evolutionary perspective, adaptive immunity only evolved as a second level of defense  against pathogens during the emergence of the agnathan vertebrate ancestors some 500 million years ago. At that stage, lymphocyte progenitors developed in organisms related to the modern hagfishes and lampreys and possibly their protochordate ancestors.^83^ Additional adaptive weaponry against intruding pathogens, such as the major histocompatibility complex, TCR and B cell receptors, only evolved in gnathostomes and became a major survival advantage of modern vertebrates.^84^

### T cells

T cells are part of the dense inflammatory infiltrate that forms in response to bacterial infection.^85^ They are the dominant cell type in the cell-mediated (macrophage/lymphocyte) immune response and are essential for both specific antibody production and polyclonal B cell activation. Based on two major surface co-receptor molecules, two functionally distinct lineages are distinguished in mature T cells, CD4 T cells and CD8 T cells. Naive CD4 T cells are activated after interaction with Ag-major histocompatibility complex II (MHC II) and polarize into specific subtypes, including T helper (Th) cells Th1, Th2, Th17 and regulatory T cells (Tregs), depending mainly on cytokine profiles.^86^ T cell activation is initiated by T cell receptor (TCR) signalling and promotes a number of signalling cascades, which lead to proliferation, cytokine production and subset differentiation.

T cell activation induces dynamic changes in miRNA expression patterns in CD4 T cell subsets. In a study designed to analyse the kinetics of activation-induced expression regulation of miRNAs in sorted CD4 and CD8 cells, the expression levels of miR-181 family, miR-17-92 clusters, miR-214, miR-146a, miR-155 and let-7 increased and the expression of miR-29, miR-125 and miR-216 decreased following T cell activation.^87^ Upregulation of miR-181 family miRNAs has been shown to enhance TCR signalling by targeting multiple phosphatases, including SH2 domain-containing protein tyrosine phosphatase 2 (*SHP2*), protein tyrosine phosphatase, non-receptor type 22 (*PTPN22*), dual-specificity protein phosphatase 5 (*DUSPS5*) and *DUDPS6*^88–90^ and enhancing the phosphorylation of immunoreceptor tyrosine-based activation motifs on the cytosolic side of the TCR/CD3 complex.^91,92^ miRNAs also affect T cell function in an indirect fashion as it has been demonstrated that upregulation of the miR-214 and miR-17-92 cluster promotes T cell activation and proliferation by targeting the negative regulator phosphatase and tensin homologue (*PTEN*) in the PI3K/Ak strain transforming (AKT) pathway.^93,94^ Another miRNA, miR-146, functions as a feedback regulator of NF-κB signalling and modulates the productive immune response, division and growth of T cells.^95^ This microRNA targets *TRAF6* and *IRAK1* for feedback control of the intensity and duration of NF-κB signalling in activated T cells.^91,95^

Imbalance in Th1 and Th2 cytokines is thought to play a role in a variety of immune-inflammatory disorders, including multiple sclerosis, arthritis, asthma, cancer^96,97^ and periodontal disease.^98,99^ Even though the Th1/Th2 model is somewhat dated in view of recent discoveries related to Th17 cells,^98^ the Th1-type phenotype is generally known as cellular and proinflammatory, whereas Th2 cells are thought to be associated with humoural immunity and feature anti-inflammatory properties.^100,101^ A number of studies suggest that miR-29 and the miR-17-92 cluster affect Th1 cell function. For example, it has been demonstrated that miR-29 inhibits Th1 cell differentiation and IFN-γ production in CD4 T cells by targeting T-box transcription factor *Tbx21* and eomesodermin (*Eomes*), two specific transcription factors for Th1 differentiation and IFN-γ mRNA expression.^102^ The miR-17-92 cluster has a similar negative effect on Th1 differentiation and IFN-γ production. However, mechanistic studies indicate that the miR-17-92 cluster targets transforming growth factor-β receptor type 2 (*TGFβRII*) and cAMP-responsive element-binding protein-1 (*CREB1*), which are involved in Treg cell differentiation, and therefore enhances Th1 differentiation.^103^ So far, three miRNAs, miR-155, let-7 family members and miR-126, have been shown to affect Th2 differentiation by regulating cytokine production. miR-155 has been demonstrated to inhibit Th2 differentiation by repressing IFN-γ signalling,^104^ whereas let-7 family members affect Th2 function by targeting IL-13 mRNA.^105^ In contrast, knockdown of miR-126 after T cell activation increased *IL-5* and *IL-13* expression and promoted Th2 differentiation through OBF.1-PU.1-GATA3 (GATA binding protein 3) signalling.^106^

The functional antagonism between Treg and Th17 cells has been proposed to be a major factor in the pathogenesis of periodontitis.^107^ After differentiation from naïve T cells, Treg cells possess a differential microRNA expression pattern that is associated with their development and function in regulating T cell responses.[108, 109] In terms of known miRNAs, miR-155 and miR-146 are highly expressed in Treg cells, whereas miR-24, miR-31 and miR-125 are found at low levels in Treg cells.^108,109^ Among these miRNAs, miR-155 directly targets *SOCS-1*, an inhibiter of STAT5 signalling, during Treg differentiation and plays a critical role in maintaining Treg lineage stability and regulatory function.^109,110^ In contrast, miR-146 represses the mRNA expression of *STAT1*, a transcription factor required for INF-γ signalling, and controls Treg-mediated suppression of Th1 responses^91,111^ Comparatively, both miR-24 and miR-31 target forkhead box P3 (*FOXP3*), a lineage specification factor of Treg cells. In related studies, downregulation of miR-24 and miR-31 has resulted in increased *FOXP3* expression and enhanced Treg differentiation and function.^112,113^ miR-125a exerts its roles in a manner partially similar to miR-24 and miR-31 to stabilize Treg lineage commitment and function. miR-125a directly suppressed several targets, including *Stat3*, *Il13* and *Ifn-g*, which are critical factors of effector lineages and detrimental to Treg differentiation.^114^

Th17 cells are involved in recruiting neutrophils and macrophages to participate in and to amplify the inflammatory reaction caused by periodontal disease.^115–117^ Th17 cell differentiation is promoted by the miR-17-92 cluster,^94^ and miR-19b, a member of the miR-17-92 cluster, represses the expression of *PTEN*, thereby augmenting the PI3K-AKT-mTOR axis essential for proper Th17 differentiation.^95^ In addition, miR-17 enhances Th17 polarization by inhibiting its target, ikaros family zinc-finger 4 (*IKZF4*).^94^ miR-17 also controls Th1 responses by targeting *TGFβRII* and *CREB1*. As a consequence, miR-17 and miR-19b suppress inducible regulatory T cell differentiation.^103^ Other miRNAs that enhance Th17 differentiation include miR-301 and miR-155. miR-301 has a negative effect on the expression of the E3 SUMO-protein ligase *PIAS3*, which suppresses the STAT3 pathway and Th17 differentiation,^118^ whereas miR-155 contributes to Th17 cell function by suppressing the inhibitory effects of Jumonji and AT-rich interaction domain-containing 2 (*Jarid2*). Jarid2 forms a complex with polycomb repressive complex 2 and targets IL-17 expression regulators, such as *Atf3*, cellular plasticity genes, such as *Tbx21* and *Eomes*, and immunoregulatory cytokine genes, such as *IL-22*, *IL-10* and *IL-9*.^119^

Previous miRNA expression profiling studies have revealed that the expression of miR-17, miR-19, miR-155 and miR-301 was upregulated in the gingival tissues of periodontitis patients and animals compared to healthy controls (Tables 1 and 2). All of these miRNAs promote the development of Th17 cells, which play an important role in the initiation and maintenance of the inflammatory response in periodontal disease. From a therapeutic perspective, inhibition of these miRNAs might shift the immune response to a Th2 profile to alter disease progression or may induce Treg cell formation to attenuate the severity of periodontal disease.

### B cells

B cells form a substantial proportion of the inflammatory infiltrate in diseased periodontal tissues, and higher levels of B lymphocyte infiltration are associated with advanced stages of periodontitis.^120,121^ Advanced periodontal inflammation also elevates the expression of five periodontal inflammation-related miRNAs that we associated with periodontal disease earlier, namely, miR-125, miR-148, miR-155, miR-181 and miR-217 (Tables 1 and 2). Indicative of a correlation between periodontal disease progression and B cell differentiation, these five miRNAs regulate the progression of B cell terminal differentiation by targeting a coordinated network of transcription factors. As an example, miR-125b is involved in the downregulation of transcription factors, B lymphocyte-induced maturation protein-1 (*Blimp1*) and interferon regulatory factor 4 (*IRF4*), to inhibit B cell terminal differentiation,^122^ whereas miR-148 has been shown to suppress BTB domain and CNC homologue 2 (*Bach2*) expression and promote plasma cell lineage commitment.^123^ miR-148 also targets proapoptotic factors *PTEN* and *Bim*, and fosters germinal centre (GC) B cell survival.^124^ miR-217 stabilizes the expression of the transcriptional repressor *Bcl*-*6* in GC B cells to increase the generation of class-switched antibodies and the frequency of somatic hypermutation by down-regulating the expression of the DNA damage response and repair gene network.^125^ Both miR-155 and miR-181 negatively regulate the expression of activation-induced cytidine deaminase (*AID*) to reinforce and repress uncontrolled AID expression.^126,127^

A summary of all relevant microRNAs, their target genes and target gene functions in immune cells and their putative regulatory role in periodontal tissues is provided in Table 3.

**Table 3:** Summary of relevant microRNAs, target genes and target gene functions in immune cells and their putative regulatory roles in periodontal tissues

Immune cells	miRNAs	miRNA in diseased tissues	Target genes	Target gene function in immune cells	Putative regulatory role of the miRNA in periodontal tissues	Ref.
Neutrophil	miR-9	Up	*NF-κ* *B1*	NF-κB family member	Feedback regulation of NF-κB pathway	36
	miR-17	Up	*ICAM-1*	Endothelial adhesion molecule	Neutrophil adhesion to endothelial cells	26
	miR-34	Up	*Dock8*	Rho family member	Neutrophil emigration	30
	miR-155	Up	*FGD4, Rac1*	Rho family member	Neutrophil emigration	30
			*SHIP-1*	Phosphoinositide lipid phosphatase	IL-8 mRNA stability	28, 29
	miR-223	Up	*CXCL2, CCL3, IL-6*	Chemoattractants	Leukocyte chemotaxis	32
			*NLRP3*	Inflammasome	Processing of inflammatory cytokines and pyroptosis	34
	miR-31	Down	*E-selectin*	Endothelial adhesion molecule	Neutrophil adhesion to endothelial cells	26
	miR-451	Down	*CPNE3, Rab5a, 14-3-3*	Binding proteins	Neutrophil chemotaxis	31
Macrophage	miR-21	Up	*STAT3*	Transcription factor in inflammation	Prevent PGE-mediated M2 generation	63
	miR-24	Up	*PKCα, CD163, CD206*	Components of pattern recognition receptor (PRR) signalling	Inhibit NF-κB activation	42
			*P110δ*	Kinase in class IAPI3 signalling	Promote M2 phenotype	64
	miR-29b	Up	*IL-6Rα, IFI30*	Components of cytokine signalling	Fine-tuning host response	43, 44
	miR-30	Up	*PKCα, CD163, CD206*	Components of PRR signalling	Inhibit NF-κB activation	42
	miR-125a	Up	*KLF13*	Transcription factor in T cell activation and inflammation	Suppress classical but promote alternative activation of macrophage	62
	miR-125b	Up	*IRF4*	Member of interferon response factor family	Enhance M1 phenotype	59, 60
	miR-142	Up	*PKCα, CD163, CD206*	Components of PRR signalling	Inhibit NF-κB activation	42
	miR-146	Up	*TRAF6, IRAK1, IRAK2*	Regulatory molecules in TLR/NF-κB pathway	Feedback regulation of inflammation	48, 49
	miR-155	Up	*MAP3K10*	Inflammatory pathway-related kinase	Endocytosis function	51
			*SOSC1*	Negative regulator of type IFN signalling	Macrophage sensitivity and tolerance	52, 53
			*BCL6*	Transcription factor counter-regulating NF-κB activity	Enhance inflammation	54
			*IL13Ra1*	Component of the type II IL-4 receptor	Promote M1 phenotype	56
			*SMAD2*	Signalling molecule of TGF-β pathway	Regulation of macrophage polarization	57, 58
	Let-7f	Up	*SOCS4, TSP-1*	Regulators of inflammation	Regulate proinflammatory macrophage activity	43, 44
	Let-7	Up	*C/EB-d*	Transcription factor in inflammatory response	Promote M2 phenotype	61
Dendritic cells	miR-24	Up	*PKCα, CD14, TLR4, MD-1*	Components of PRR signalling	Inhibit NF-κB activation	82
	miR-30	Up	*PKCα, CD14, TLR4, MD-1*	Components of PRR signalling	Inhibit NF-κB activation	82
	miR-126	Up	*TSC-1*	Negative regulator of mTOR	Survival and function of plasmacytoid DC	79
	miR-142	Up	*PKCα, CD14, TLR4, MD-1*	Components of PRR signalling	Inhibit NF-κB activation	82
	miR-155	Up	*c-FOS, PU.1*	Transcription factors	DC maturation	73
			*SOCS-1*	Negative regulator of IL-2 signalling	DC maturation and pathogen binding	71
	let-7	Up	*SOCS-1*	Negative regulator of IL-2 signalling	Enhance a proinflammatory phenotype of mature DC	74, 75
T cells	miR-17	Up	*IKZF4*	Member of the Ikaros family transcription factors	Promote Th17 phenotype	95
			*TGFβRII*	TGF-β signalling	Enhance Th1 differentiation and prevent Treg response	104
			*CREB1*	Transcription factor regulating FoxP3 expression	Enhance Th1 differentiation and prevent Treg response	104
	miR-19	Up	*PTEN*	Negative regulation of PI3K-AKT-mTOR axis	Enhance Th17 polarization	95
	miR-24	Up	*FoxP3*	Lineage-specific factor of Treg cells	Inhibit Treg differentiation	113, 114
	miR-29	Up	*TBX21EOMES*	Transcription factors for INF-γ	Inhibit Th1 differentiation	103
	miR-31	Up	*FoxP3*	Lineage-specific factor of Treg cells	Inhibit Treg differentiation	113, 114
	miR-125a	Up	*STAT3, IL-13, INF*-γ	Critical factors for effector lineages	Inhibit Treg differentiation	115
	miR-146	Up	*TRAF6, IRAK1*	Regulatory molecules in TLR/NF-κB pathway	Feedback regulation of NF-κB signalling in T cells	92, 96
			*STAT1*	Transcription factor for INF-γ	Suppress Th1 response	112
	miR-155	Up	*SOCS-1*	Inhibitor of STAT5 signalling	Maintain Treg homeostasis and function	104
			*INF-γRA*	Cytokine receptor	Inhibit Th2 differentiation	105
	miR-181	Up	*SHP2, PTPN22, DUSPS5, DUSPS6*	Phosphatases	Elevate steady-state levels of phosphorylated interaction and reduce T cell receptor signalling threshold	89, 90
	miR-301	Up	*PIAS3*	Inhibitor of STAT3 pathway Transcription factors for plasma cell differentiation	Promote Th17 response	119
	let-7	Up	*IL-13*	Cytokine	Inhibit Th2 differentiation	106
	miR-214	Down	*PTEN*	Negative regulator of AKT signalling	Enhance Th17 polarization	94
B cells	miR-125b	Up	*Blimp1, IRF4*	Transcription factors for plasma cell differentiation	Promote B cell diversification in GC	123
	miR-148	Up	*PTEN, Bim*	Proapoptotic factors in B cells	Promote plasmablast survival	125
			*Bach2, Mitf*	Transcription factors for plasma cell differentiation	Promote plasma cell differentiation	124
	miR-155	Up	*AID*	A potent DNA mutator	Antibody affinity maturation	127
	miR-181	Up	*AID*	A potent DNA mutator	Antibody affinity maturation	128

## miRNAs as biomarkers for periodontal disease

At present, the most common oral fluid-based molecular biomarkers for periodontitis fall into three general categories: (i) host-derived enzymes and their inhibitors, (ii) inflammatory mediators and host response modifiers and (iii) tissue breakdown products.^128–131^ These molecular biomarkers detect the presence and severity of disease, but cannot predict the initiation and early transition of periodontal disease. Most recently, miRNAs have emerged as a novel class of highly sensitive and specific biomarkers.^132^ miRNAs remain stable in extracellular fluids due to their packaging^133–135^ and are thus ideally suited to serve as non-invasive biomarkers for periodontal disease.

Previous studies have demonstrated that miRNAs affect the host immune response by regulating the expression of downstream target genes. In periodontal disease, oral bacteria and inflammatory conditions affect the function of oral epithelial and immune cells and dysregulate miRNA expression in these cells. While immune cells and non-immune cells synthesize miRNAs intracellularly, they also actively release miRNAs into extracellular environments, including extracellular fluids.^136–138^ The released miRNAs are associated with RNA-binding proteins or high-density lipoproteins, or they are enclosed within lipid vesicles. They are highly stable in extracellular fluids.^139^ Regardless of their biological functions, five miRNAs, including miR-142-3p, miR-146a, miRNA-155, miR-203 and miR-223, have been proposed as markers for periodontal disease.^140^

Gingival crevicular fluid (GCF) and saliva have been considered to be ideal sampling environments for periodontal diagnosis because of their proximity to the periodontium and due to their suitability for non-invasive sample collection.^141,142^ GCF is an exudate of periodontal tissues, which is located in the gingival sulcus of healthy subjects or in the periodontal pocket of periodontitis patients. miRNA profiling in GCF of healthy subjects or periodontitis patients has resulted in the identification of more than 600 miRNAs. Among these, miR-223 was the most highly expressed miRNA in GCF samples, and this miRNA was also upregulated in periodontitis samples.^143^ In addition, a subset of miRNAs was altered in the GCF of periodontal disease samples and therefore holds potential as a biomarker for periodontitis.^143^ Compared to GCF, saliva is more complex as it contains not only GCF but also salivary gland products, oral mucosal exudates and a potential pool of biological markers in a hypotonic environment. The concept of salivaomics has been proposed, which encompasses genomics, transcriptomics, proteomic, metabolomics and miRNA profiles.^144,145^ miRNA profiling in saliva has been investigated as a diagnostic approach for oral cancer, pre-cancer and oral lichen planus disease.^146^

Further studies of miRNAs in GCF or saliva and their relationship with periodontal diseases may provide novel diagnostic tools for the early detection of periodontal disease and treatment plan monitoring.

## miRNA targeting as a potential therapeutic strategy for the treatment of periodontal disease

In recent years, novel miRNA therapeutics have gained increasing prominence as potential therapeutic agents because of their small size, their ability to easily pass through tissues and membranes, their microregulatory effects on gene expression and their ability to affect multiple and related mRNAs at the same time.^1^ Early clinical successes for miRNA applications in the treatment of disease include a mimic for the tumour suppressor miRNA miR-34 and anti-miRs targeting miR-122 for the treatment of hepatitis.^147^ The complexity of periodontal disease and clinical applicability of miRNA mimics and anti-miRs to control the miRNA environment of host tissues and disease processes are related to their potential use in the treatment of periodontal disease.

One obvious miRNA targeting opportunity is presented in form of the altered host miRNA expression profile in periodontal tissues of periodontitis patients and in animals suffering from periodontal disease (Tables 1 and 2) as it is triggered by oral microbial infections. There are two basic mechanisms involved in the alteration of miRNA expression upon infection with oral pathogens, (i) pathogen-encoded miRNAs designed to mimic host miRNAs to aid pathogen reproduction and survival and (ii) changes in host miRNA expression levels as part of the host immune response.^5,148^ Loss of the homeostatic balance of miRNA regulation affects host immune responses to pathogens and benefits bacterial infection. Therefore, manipulating miRNA function to modulate exuberant inflammation associated with tissue breakdown may have therapeutic potential for the treatment of periodontal disease. Potential strategies for miRNA targeting aim to either augment or dampen the expression of specific miRNAs, depending on the biological or pathological disposition of the miRNA(s). miRNA levels may be either enhanced or limited by using miRNA mimics or miRNA antagonists, respectively. miRNA mimics are chemically synthesized short double-stranded oligonucleotides that functionally mimic a pre-miRNA complex.^149,150^ In contrast, miRNA antagonists are single-stranded oligonucleotides that complement miRNA sequences and are designed to functionally reduce miRNA activity.^151,152^

miRNA regulation of gene expression as part of the immune response to infection is a highly complex process. Not only do unique bacteria regulate different host miRNAs, but the same bacterium may also cause different miRNA responses to infection in different cell types.^5,153^ However, genome-wide miRNA transcriptional response analysis in human immune cells responding to various bacteria has resulted in the identification of a set of core miRNAs in response to bacterial infection.^154^ For example, miR-155, miR-146, miR-125, miR-21 and let-7 are commonly affected miRNAs during bacterial infection.^15,153^ Especially, changes in miR-155 expression are part of the inflammatory immune response of various immune cells in both innate and adaptive immunity.^155^ The presence of common miRNA profiles as part of the cellular immune response to infection suggests that manipulating the expression of these core miRNAs may alter or reverse the progression of periodontal disease.

Although therapeutic targeting of miRNAs for the treatment of periodontal disease is still in its infancy, convincing proof of principle is provided by studies of immune diseases and tumour immune responses in animal models. Immune responses driven by activated T cell subsets are involved in the development of pathologic immune disorders. For example, Th1 cells promote cellular immunity and affect the development of autoimmune diseases, while Th2 cells mediate humoural immunity and trigger allergic immune responses.^156^ Addressing the Th2 cell-mediated allergic immune response, intranasal application of miR-126 or miR-145 antagonists suppressed the effector function of Th2 cells and inhibited house dust mite-induced allergic airway diseases in animal lungs.^106,157^ Similarly, miR-106 and let-7 family members are differentially expressed in allergic murine models. Antagonism of miR-106 or overexpression of let-7 modulated the Th2 response in airways and attenuated the development of asthma symptoms.^105,158^ Th17 T cells are key players in various autoimmune diseases in humans and animal models, including experimental autoimmune encephalomyelitis (EAE). In earlier studies, it has been demonstrated that Th17 cell differentiation is regulated by miR-326. *In vivo* silencing of miR-326 using a lentiviral approach inhibited Th17 differentiation and attenuated EAE in mice.^159,160^ In addition, overexpression of miR-10 by retroviral vectors inhibited the conversion of inducible Treg cells into follicle Th cells and limited differentiation into the Th17 subset of Th cells. Thus, miR-10 supplementation resulted in a protective effect and attenuated the onset of EAE.^160,161^

miRNA-based immune responses have also been investigated as potential targets for anti-tumour immunotherapies. In a preclinical study, miR-138 was selected as a candidate for modulating immune checkpoint activity in murine models of GL261 glioma.^162^ Specifically, intravenous delivery of miR-138 liposomal complexes resulted in decreased intratumour Fox3+ regulatory T cells and inhibited the expression of cytotoxic T-lymphocyte-associated molecule-4 (*CTLA-4*) and programmed cell death-1 (*PD1*) in CD4+ T cells, two important checkpoints in tumour progression.^162^ Augmentation of miR-138 *in vivo* suppressed the growth of glioma cells and prolonged the survival of the animals suffering from glioma.^162^ In another study, nanoparticles with miR-155 mimics as cargo were delivered into DCs *in vivo* to enhance the miR-155 activity in an ovarian cancer microenvironment,^163^ resulting in a transformation of DCs from immunosuppressive to immunostimulatory cells and an activation of potent anti-tumour immune responses.^163^

Together, these clinical and preclinical studies illustrate the enormous therapeutic potential of miRNA-based strategies. Therapeutic modalities for the treatment of periodontal disease may include the application of miR-based therapeutics in combination with current periodontal treatment approaches. Future research will identify unique combinations and dosages of miRNAs in combination with suitable carrier vehicles and delivery strategies to optimally combat and reverse periodontal disease.
